# Chebifier: automating semantic classification in ChEBI to accelerate data-driven discovery

**DOI:** 10.1039/d3dd00238a

**Published:** 2024-03-26

**Authors:** Martin Glauer, Fabian Neuhaus, Simon Flügel, Marie Wosny, Till Mossakowski, Adel Memariani, Johannes Schwerdt, Janna Hastings

**Affiliations:** a Otto von Guericke University Magdeburg Germany; b Institute for Implementation Science in Health Care, University of Zurich Switzerland janna.hastings@uzh.ch; c School of Medicine, University of St. Gallen Switzerland; d Swiss Institute of Bioinformatics Switzerland; e University of Applied Sciences Merseburg Germany

## Abstract

Connecting chemical structural representations with meaningful categories and semantic annotations representing existing knowledge enables data-driven digital discovery from chemistry data. Ontologies are semantic annotation resources that provide definitions and a classification hierarchy for a domain. They are widely used throughout the life sciences. ChEBI is a large-scale ontology for the domain of biologically interesting chemistry that connects representations of chemical structures with meaningful chemical and biological categories. Classifying novel molecular structures into ontologies such as ChEBI has been a longstanding objective for data scientific methods, but the approaches that have been developed to date are limited in several ways: they are not able to expand as the ontology expands without manual intervention, and they are not able to learn from continuously expanding data. We have developed an approach for automated classification of chemicals in the ChEBI ontology based on a neuro-symbolic AI technique that harnesses the ontology itself to create the learning system. We provide this system as a publicly available tool, Chebifier, and as an API, ChEB-AI. We here evaluate our approach and show how it constitutes an advance towards a continuously learning semantic system for chemical knowledge discovery.

## Abstract 2

Connecting chemical structural representations with meaningful categories and semantic annotations representing existing knowledge enables data-driven digital discovery from chemistry data.

## Graphical abstract

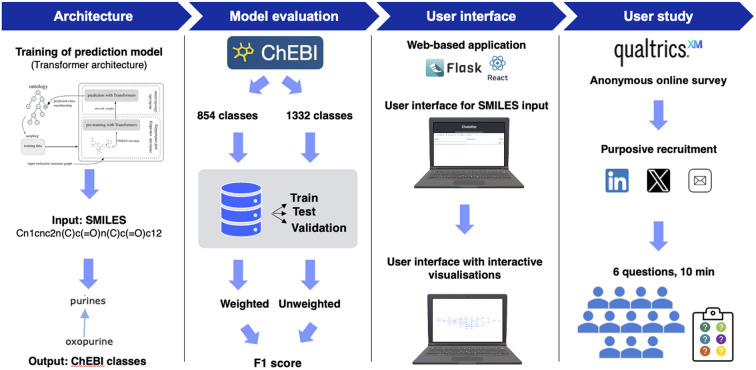

## 1 Introduction

Data-driven discovery is accelerating scientific research across domains such as materials design,^1^ drug discovery,^2^ pharmacogenomics^3^ and metabolism.^4^ Harnessing chemical data for digital discovery requires the possibility to connect information-rich chemical structures with semantic annotations representing prior knowledge. Knowing which classes a molecule belongs to, helps to predict its chemical behaviour and uses, as well as enrich data and drive discovery approaches. Ontologies are exemplary semantic annotation resources which provide definitions and a classification hierarchy for a domain.^5^

ChEBI,^6,7^ or Chemical Entities of Biological Interest, offers a large and widely used ontology in the domain of life sciences chemistry. It is a manually developed ontology that contains (as of February 2024) 61 189 fully curated entries associated with chemical and biological knowledge. For example, ChEBI contains the entity *caffeine* (CHEBI:27732) which is classified chemically as a *purine alkaloid*, and biologically as an *environmental contaminant*, a *phosphoric diester hydrolase inhibitor*, and a *food additive*. ChEBI is the foremost chemical ontology in use for life science applications and for annotating FAIR data for data exchange and integration on the Semantic Web.^8^ The manual curation of ChEBI by experts is a prerequisite for its success, since it enables ChEBI to capture the nuances of chemical terminology, and represent expert knowledge about chemicals. However, manual maintenance poses limits to how quickly the ontology can grow. The manually curated portion of ChEBI only grows at a rate of approximately 100 entries per month while, for example, the PubChem database,^9^ an important open repository of life science chemicals that is not manually curated, contains 110 million chemicals. The size of PubChem can be taken as a rough estimate of the number of (at least potentially) biologically interesting chemicals, which vastly outstrips the number that can be manually annotated in ChEBI. These numbers illustrate the limitations of a fully manual curation process and the need for some form of automation. For that reason, as we have argued previously,^10–12^ automated approaches to support the ontology development and application are desirable. At the same time, fully automated approaches are unlikely to match the nuance and sophistication of human annotators, and in particular, cannot replace the community-involving consensus-building work of negotiating contested classifications.^13^

In this paper we describe an approach to automatically classify chemicals in ChEBI, that learns from the existing ontology by training a machine learning model that is able to then extend the ontology with novel structures. We have made our approach available in the web-based tool Chebifier, a tool that automatically classifies a given list of chemical entities with classes from ChEBI. As we will discuss in Section 2, the classification is done *via* a Transformer model that is trained solely based on the information contained in ChEBI. Chebifier may be useful for anybody interested in data-driven discovery involving meaningful interpretation of chemicals based on chemical structures. In addition, it may be used to automatically generate extensions of ChEBI with chemicals that have not yet been included in the ontology, or be used in a semi-automatic approach by the ChEBI curation team to provide suggested classifications, and, thus, improve the speed of the manual curation process. Since Chebifier is itself trained from the content of ChEBI, future extensions of ChEBI will improve the performance of Chebifier. Thus, in the long run, our approach has the potential to create a virtuous feedback cycle of ontology extension, checking and then improvement of the classification model, that serves to lay the foundations for semantic chemical discovery research.

## 2 Methods

The underlying strategy of our approach is that we use the data from the ontology itself to train a neural network that is then able to mimic the choices of the chemical curators who developed the existing ChEBI ontology structure. An advantage of our approach is that it does not require developers to maintain a set of explicit rules in addition to the ontology, since the neural network learns classification rules during training. Furthermore, as the ontology changes and grows over time, the neural network may be retrained and automatically adjusts to changes and benefits from improvements of the ontology (see Section 3.2).

We have developed this approach over the past years (see, *e.g.*, Glauer *et al.*^10,14^) and here present a significant enhancement on our previous work through both an extension to a significantly larger number of classes, requiring introduction of a novel weighting scheme, as well as the development and presentation of a web interface making the model accessible to end users, which was tested in a user study.

In the following subsections we will present the architecture of Chebifier, its prediction model, its user interface, and the methods we used to conduct the user study.

### 2.1 Architecture

Chebifier is developed as a prediction model based on input chemical structure representations in SMILES (Simplified Molecular Input Line Entry System)^15^ format, a widely used string representation for chemical structures. The SMILES format encodes molecules as semi-readable sequences of characters that represent atoms and bonds, which are efficient for storage, exchange and computational processing. For example, the SMILES for *caffeine* is 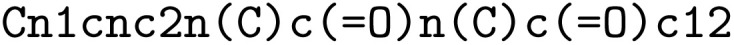.

Our prediction model is based on a Transformer architecture. Transformers require their inputs to be compiled into a set of tokens (which are in turn embedded into a high-dimensional latent space). Thus, one of the key parameters of the approach is the choice of encoding strategy. The simplest approach to the encoding would be to consider SMILES merely as a straightforward sequence of characters. However, this naïve approach to encoding can lead to the loss of vital chemical context. For example, consider the molecule represented by 
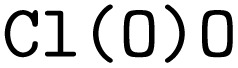
. If we break this down into individual characters, we get “C”, “l”, “(”, “O”, “)”, “O”. While this representation is appropriate for general string manipulation, it fails to capture the chemical significance of the specific components. In this case, “Cl” should be considered as a single token because it represents a chlorine atom, a chemically meaningful unit. At the same time, “C” often refers to carbon in other molecules. Thus, faced with only the sequence of characters in a naïve encoding, the model would need to learn to differentially interpret the characters depending on their contexts, which would further complicate an already challenging training task. Therefore, we decided to pre-process the input strings to create chemically feasible tokens, treating atom labels and any associated charges as single entities rather than isolated individual characters. This ensures that the representation remains faithful to the underlying molecular structure and facilitates more accurate chemical analysis and processing.

This pre-processed data then serves as the input to a prediction model. For this purpose, we developed the ChEB-AI framework††https://github.com/ChEB-AI/python-chebai – a general-purpose deep learning framework for ontology-based training tasks in chemistry. In previous research,^10–12^ we have analysed the performance of different architectures on the ontology extension task. The pipeline used for the tool presented in the current work is based on an ELECTRA model,^16^ a Transformer-based deep learning model that we found to be the most performant architecture. Transformers are modern deep learning architectures that have demonstrated noteworthy predictive performance across a range of different tasks. These models use an attention mechanism^17^ to contextualise the information flow within the model. In addition, the attention mechanism enables a form of transparency about how the model processes information and allocates attention across different parts of the input data. In our previous study,^10^ we observed that the attention mechanism can be used to obtain interpretable visualisations that highlight structures used by the model when it is computing the predicted classifications.

For the pipeline we describe in the current manuscript, we pre-train the ELECTRA model on a dataset of extracted molecules from the PubChem database and then fine-tune the model on two different datasets extracted from ChEBI.

The original dataset we used in our previous research in which we established the appropriate architecture for the chemical ontology prediction problem was limited to a selection of 500 classes to predict.^10^ For the present manuscript, we aimed to go beyond our earlier feasibility demonstration and implement a pipeline that was maximally useful in practice. Thus, we substantially expanded the number of classes included in the prediction model to the full list of classes that met the selection criteria that ensured that each of the selected classes had a minimum number of members, *i.e.*, subclasses with annotated SMILES strings. For our earlier work, we used a selection threshold of 100 members. Consequently, we introduced the *ChEBI*^854^_v200_ dataset, which encompasses 854 classes as labels, which corresponds to all subclasses of *chemical entity (CHEBI:24431)* in ChEBI (as of ChEBI release 200) that have at least 100 subclasses with annotated SMILES strings. Chemical and biological roles are not yet part of this classification.

Note that, as will be discussed further in Section 4.1 below, due to the volume of submissions it receives, ChEBI already uses some automation strategies to pre-classify content prior to full manual curation. However, the released ontology contains a mix of manually curated and partially automated content, which is then included in our training data. While it would be better to work with a completely manually checked sub-set of ChEBI, the way that the ontology is built precludes obtaining a fully connected ontology graph from a manually checked sub-set.

Chebifier 1.0 was based on a model trained on *ChEBI*^854^_v200_. As we will elaborate on subsequently, input from our user study indicated a desire for including more specific classes from ChEBI. In response, we created the *ChEBI*^1332^_v200_ dataset, which includes all classes in ChEBI (release 200) that have a minimum of 50 SMILES-annotated subclasses. The reduction in the size of members required allowed the dataset to expand to a wider range of classes, resulting in 1332 class labels that the model is able to predict. Chebifier 1.1 uses a model that is trained on *ChEBI*^1332^_v200_.

**Fig. 1:**
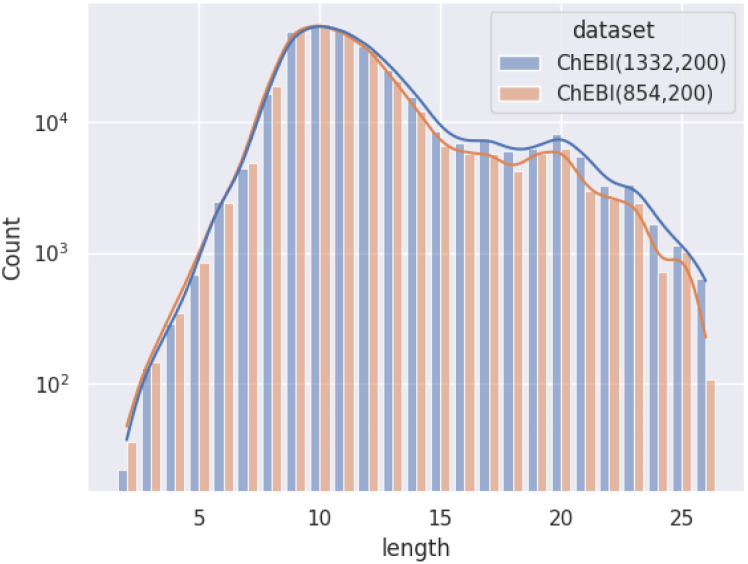
Distribution of path lengths (log scale) amongst non-redundant superclasses among molecular entities in the test set.

**Fig. 2:**
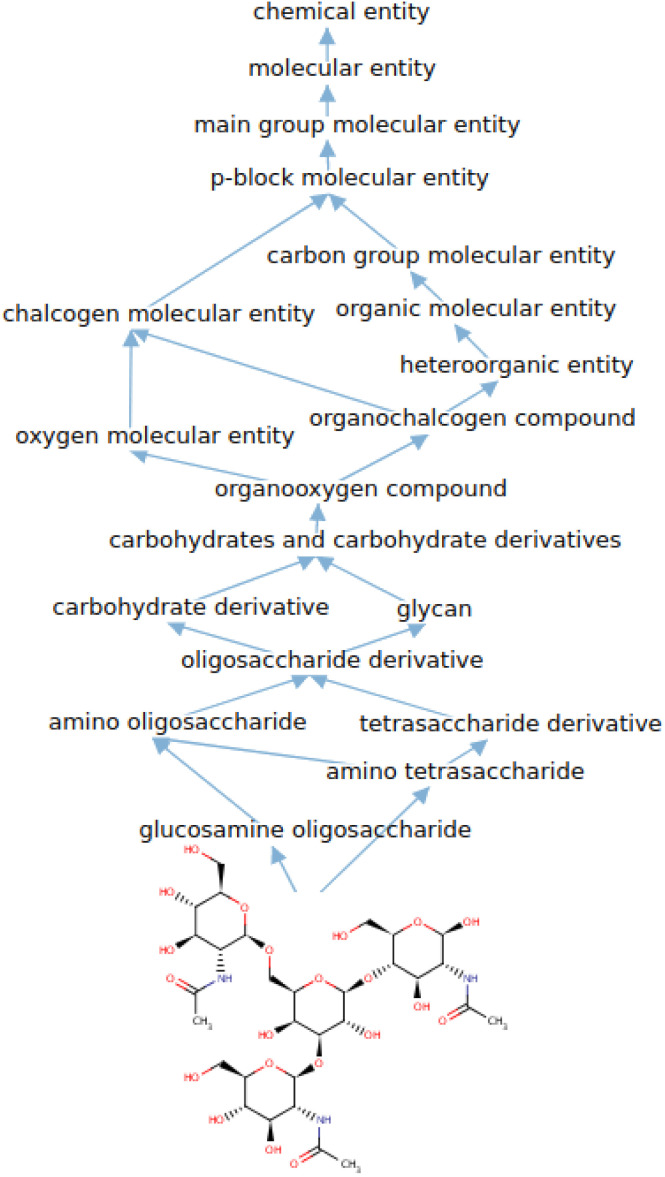
Example of subsumption paths from CHEBI:71342 to CHEBI:24431 (chemical entity). CHEBI:71342 has two direct parents and there are nine paths of length 12 and nine paths of length 14 from them to CHEBI:24431.

Fig. 1 illustrates the difference between *ChEBI*^854^_v200_ and *ChEBI*^1332^_v200_ by comparing the distribution of path lengths within the test set. These paths have been calculated based on the subsumption graph from each direct parent in the dataset, *i.e.*, those that cannot be inferred from any other superclasses. It can be seen that when passing from *ChEBI*^854^_v200_ to *ChEBI*^1332^_v200_, the focus of the dataset shifts towards classes deeper down in the hierarchy. The way paths are counted is illustrated in Fig. 2.

Preliminary tests indicated that the increased number of labels had a negative impact on the prediction quality for smaller classes. Therefore, we introduced an additional weighting scheme proposed by Cui *et al.*^18^ that penalises prediction error based on the number of members in each class *C*. This weighting mechanism assigns each class *C* of size |*C*| an *effective number*
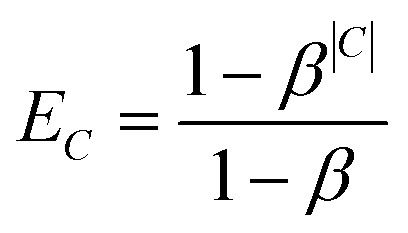
, which is an estimate of the space that a set of |*C*| possibly overlapping volumes of size 1 would occupy. The intuition behind the metric is that new instances to already large classes have a smaller impact on the overall size than it would have for a smaller class. As proposed by Cui *et al.* we used the inverse of this *effective number* as a weighting with *β* = 0.99:
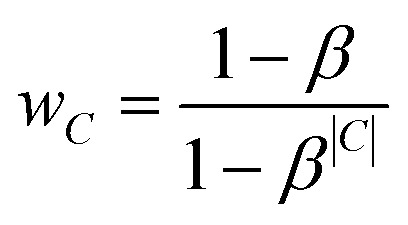


The datasets *ChEBI*^854^_v200_ and *ChEBI*^1332^_v200_ are both based on version 200 of ChEBI. While ChEBI is continuously updated and newer versions of ChEBI are available (as of November 2023, the newest version is v237), we used version 200 in our experiments reported here in order to be able to compare the results to the previous Chebifier version.

Chebifier can be automatically updated for new ChEBI versions, and the objective of our work is to keep the model up to date with the latest version of the database regularly in the future. The fine-tuning of a single model takes less than 15 hours on two NVIDIA TITAN X's. Considering ChEBI's monthly update schedule, it is thus possible to implement an automated update mechanism that pulls a new version of the ontology and updates the model automatically. However, automated quality assurance would be necessary to guarantee future high-quality predictions.

In order to evaluate the effect of changing versions, we use *ChEBI*^709^_v148_, *i.e.*, a dataset comparable to *ChEBI*^854^_v200_ derived from ChEBI-version 148, approximately 4 years before version 200. In order to get comparable results, we have first split the *ChEBI*^854^_v200_ dataset into a training, validation and test set (see Section 2.2). Then we have created *ChEBI*^709^_v148_, encompassing the 709 classes in ChEBI version 148 that have at least 100 subclasses with annotated SMILES strings. Importantly, we have not done an independent random split into training, validation and test sets for *ChEBI*^709^_v148_. Instead, we used the test set from *ChEBI*^854^_v200_ and reduced the labels to those that were represented in ChEBI version 148. This means that the SMILES-annotated classes in the *ChEBI*^854^_v200_ test set are excluded from the *ChEBI*^709^_v148_ training and validation sets. Therefore, we are able to train models on training and validation sets from both ChEBI versions separately but evaluate them on the same test set. Some subsumption relations were removed from ChEBI between version 148 and version 200. This caused a total of eight classes in our dataset to drop below the member threshold for label selection. Therefore, only 701 of 709 classes in *ChEBI*^709^_v148_ are also part of *ChEBI*^854^_v200_.

### 2.2 Model evaluation

For our evaluation, we split the *ChEBI*^854^_v200_ and *ChEBI*^1332^_v200_ datasets into a test (12.75%), training (85%), and validation set (2.25%). Since the test set for *ChEBI*^709^_v148_ is derived from the one used for the previous *ChEBI*^854^_v200_ and *ChEBI*^1332^_v200_, we only split the remaining dataset into a training (97.75%) and validation set (2.25%). The primary metric we used for our evaluation was the F1 score. To address the imbalance in class sizes in both datasets, we compared two different strategies for aggregation of our scores. The *micro*-aggregation method averages over the F1 scores of individual datapoints. This metric gives an insight into the prediction quality for a given molecule and is widely used in deep learning applications. However, this metric treats all class labels are equally valuable, but the hierarchical nature of the labels in ChEBI datasets does not fit that assumption: a correct prediction of classes higher up in the hierarchy is often easier for the model and also less interesting, therefore higher-up predictions should ideally be ranked lower by the metric. At the same time, classes higher up in the hierarchy are also much more frequently labelled, which skews the micro F1 score in their favour. To address this, we also considered the *macro*-aggregation method in our evaluation, which aggregates the F1 scores label-wise. This puts a higher penalty on prediction models that disregard infrequent or hard-to-predict classes.

Since the reason for training a model on *ChEBI*^1332^_v200_ was to achieve more specific classifications, we also evaluate it by comparing the specificity of its predictions compared to *ChEBI*^854^_v200_. It is not straightforward to define specificity of a prediction, as there are different ways that specificity can be defined. For our evaluation we consider several different approaches: (1) we compare path lengths between predicted parents and directly asserted parents, (2) we compare path lengths between predicted parents and the ontology root class, and (3) to directly compare the two versions of the model, we determine for each prediction by the *ChEBI*^854^_v200_ model on our test set, whether the *ChEBI*^1332^_v200_ model predicts a more specific class, the same class, or a less specific class.

### 2.3 User interface

**Fig. 3:**
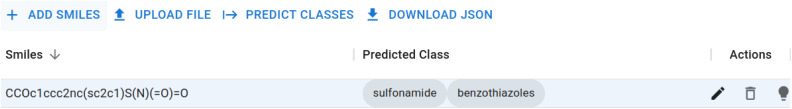
The user interface of Chebifier with example input. The submitted string represents the class of *ethoxzolamide* (CHEBI:101096). The predicted superclasses match those documented in the ChEBI ontology.

The Chebifier user interface is a web application developed based on a simple Python Flask server with a JavaScript React front-end. Users can upload a batch of SMILES strings or input them manually. The input is then compiled into a request and submitted to our processing server. The calculated predictions are then returned and displayed next to the input form. The interface used for this process is depicted in Fig. 3.

**Fig. 4:**
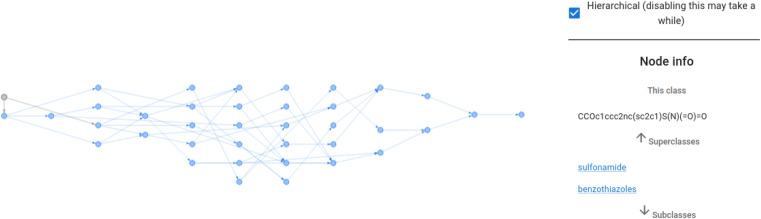
The ontology fragment for *ethoxzolamide* as predicted in Fig. 3. The interactive visualisation displays class labels when the pointer hovers over a node.

Additionally, the front-end visualises an interactive graph as shown in Fig. 4. This interactive graph depicts an extended version of ChEBI that contains the submitted SMILES strings as new classes, and shows the predicted parents and paths to the root of the ontology in a dynamic visualisation in which the class labels are displayed when each node is selected. Furthermore, there is a details page for each submitted molecule that displays its molecular graph (rendered in RDKit^19,20^), its predicted position in ChEBI, and a visualisation of the attention weights that the model used to reach this prediction.

### 2.4 User study

An anonymous online user survey in the English Language was conducted from June to August 2023 using Qualtrics (https://www.qualtrics.com). Following a purposive recruitment strategy, we engaged participants *via* LinkedIn, Twitter, and direct emails. The recruitment strategy included invitations to the curators of ChEBI, the PubChem team, the developers of key partner databases such as Rhea,^21^ and members of the chemical informatics community who had previously shown an interest in chemical classification.

The objectives of the user study were threefold. First, we aimed to assess user satisfaction with the accuracy and exactness of the automated classifications. Second, we aimed to gather insights on the user interface, including associated visualizations. Third, we aimed to explore the acceptability and reception of a machine learning-based application for the problem of predicting structure-based chemical ontology classifications.

The survey questionnaire was divided into two parts, collectively comprising a total of six questions with open-text response options. It was estimated that respondents would require approximately 10 minutes to complete the survey. The initial part of the survey directed participants to engage with the Chebifier tool and add one or more SMILES strings representing molecular entities of their choice, and then execute class prediction. Once the prediction was returned, respondents were asked to rate its correctness and appropriateness. Subsequent to the prediction step, participants were directed to open the individual details page for a single prediction. On this page, a visualization of the molecular structure of the input SMILES was displayed along with a subset of ChEBI, relevant to the molecule's direct parents. Finally, the prediction model's internal attention parameters were displayed. Participants were asked to evaluate the utility of the visualization representing the subset of the ChEBI hierarchy. Furthermore, they were encouraged to explore the attention clusters, encompassing various heads and layers, and asked for recommendations for improvement. Concluding the survey, respondents were offered an opportunity to provide further suggestions to enhance the Chebifier tool as a whole.

## 3 Results

The API is implemented as open source software available from the GitHub repository at https://github.com/ChEB-AI/. The Chebifier web tool is available at https://chebifier.hastingslab.org/.

### 3.1 Model evaluation

Two different metrics were used in the evaluation to comprehensively assess the performance of all models. The details of these evaluation metrics are explained in Section 2.2. When comparing F1 scores (Table 1), both weighted and unweighted labels were considered for both datasets. This comparative analysis provided insight into how well the models handle the heterogeneous distribution of classes on both datasets. Micro F1 scores, which indicate overall precision and recall, showed similarities in both datasets. This metric provides a measure of how well a the model performs given an individual chemical entity. The macro F1 scores, while lower than the micro F1 scores, provide insight into the model's performance on the overall class level. The lower macro F1 scores can be attributed to the inherent tendency of the model to focus more on predominant classes, a challenge which mirrors the broader problem of bias in machine learning more generally.

**Table 1:** Comparison of F1 scores on both datasets with different aggregation methods. The best result for each combination of dataset and aggregation method is highlighted in bold

	Micro		Macro	
	Unweighted	Weighted	Unweighted	Weighted
*ChEBI* ^854^_v200_	**0.9032**	0.8901	0.6070	**0.6372**
*ChEBI* ^1332^_v200_	**0.9020**	0.9010	0.6022	**0.6552**

**Fig. 5:**
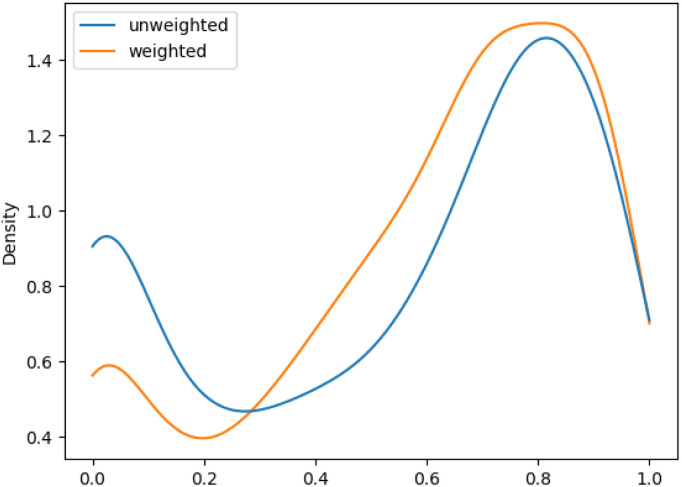
Distribution of F1 scores for among classes on weighted and unweighted *ChEBI*^854^_v200_.

**Fig. 6:**
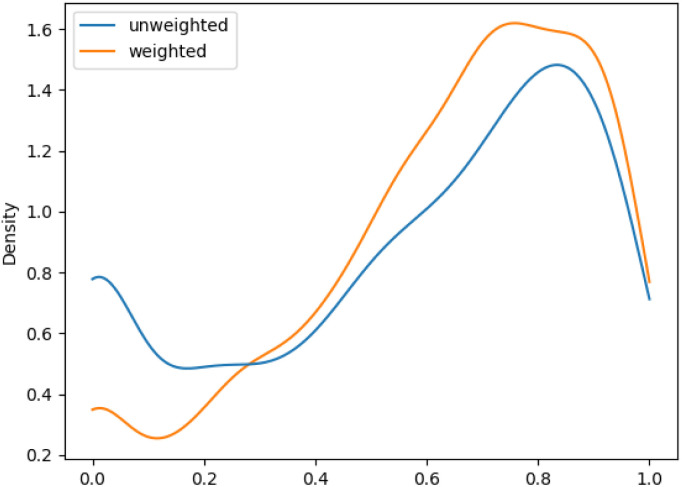
Distribution of F1 scores among classes on weighted and unweighted *ChEBI*^1332^_v200_.

We aimed to mitigate this tendency by introducing additional weighting, which would allow for a more balanced assessment, especially for datasets with unbalanced class distributions. The evaluation metrics showed an increase in performance of the macro F1 evaluations with additional weighting. This improvement was more pronounced for *ChEBI*^1332^_v200_ compared to *ChEBI*^854^_v200_, with the former benefiting more due to its hierarchical structure with a larger number of classes further down the hierarchy. Fig. 5 and 6 show the density plot of the distributions of F1 scores for both datasets with and without weighting. It can be seen that the weighting improved predictions in both cases and lowered the number of cases in which no correct predictions were made.

### 3.2 Comparison between ChEBI versions

ChEBI is continuously improved by manual curation. A benefit of Chebifier is that it can be automatically adjusted to new ChEBI versions. Since subsequent versions introduce additional entities to the ontology, we expect that this increases the classification performance for classes for which Chebifier has more samples to learn from.

To analyse the effect of version changes in ChEBI, we compare classification performance for two datasets derived from different versions, namely version 148 from February 2017 and version 200 from January 2021.‡‡These and other historical ChEBI versions are available at https://bioportal.bioontology.org/ontologies/CHEBI For both versions, we have selected classes with at least 100 SMILES-annotated subclasses as labels, resulting in the two datasets *ChEBI*^709^_v148_ and *ChEBI*^854^_v200_.

Notably, both datasets share the same test set. This is ensured by first creating a train-validation-test split for *ChEBI*^854^_v200_, and afterwards creating a train-validation split for *ChEBI*^709^_v148_, discarding all SMILES-annotated classes that are present in the *ChEBI*^854^_v200_ test set. Therefore, this test set can be used both for models trained on *ChEBI*^854^_v200_ and for models trained on *ChEBI*^709^_v148_. For the evaluation of *ChEBI*^709^_v148_ models, only the labels of the test set have been changed to the 709 labels present in *ChEBI*^709^_v148_.

Table 2 shows the different dataset sizes and the classification performance for both versions. After 4 years of development, more classes are above the threshold of having at least 100 subclasses that are associated with SMILES. Thus, *ChEBI*^854^_v200_ contains 145 more classes than *ChEBI*^709^_v148_. Further, ChEBI version 200 is able to yield more than 50% additional training data. This results in an overall higher F1 score, both with micro- and macro-aggregation.

**Table 2:** Comparison of number of classes, training size and F1 scores with different aggregation methods for *ChEBI*^709^_v148_ and *ChEBI*^854^_v200_. The macro F1 score was calculated for all classes predicted by the model and, for comparison, also on the subset of 701 classes that appear in both ChEBI version. The best result for each score is highlighted in bold

	Classes	Training set size	Micro F1 (all cl.)	Macro F1 (all cl.)	Macro F1 (common)
v148	709	80 639	0.8546	0.5133	0.5200
v200	854	129 187	**0.9032**	**0.6070**	**0.6231**

The significance of comparing the macro F1 scores between the models is limited by the fact that *ChEBI*^709^_v148_ and *ChEBI*^854^_v200_ contain different classes. (For example, the improvement of the macro F1 score between version 148 and version 200 score could be an effect of version 200 containing classes that are easier to learn.) Thus, to enable a better comparison we also calculated the macro F1 scores on the 701 classes that are common to *ChEBI*^709^_v148_ and *ChEBI*^854^_v200_. With respect to these shared classes, the macro F1 score is 10% higher for the model trained on version 200.

The results in Table 2 show that the improvements to ChEBI over time lead to significant improvements of the trained model, both with respect to performance as well as class coverage. Even though the task of predicting more individual classes is in general more difficult, this effect seems to be outweighed by the improvement to the ontology over time and the additional available training data.

The result illustrates a benefit of our approach, which enables us to convert improvements and extensions of the underlying ontology into improvements of the trained model and thereby create a positive feedback loop towards a continuous semantic learning cycle.

### 3.3 User study

**Fig. 7:**
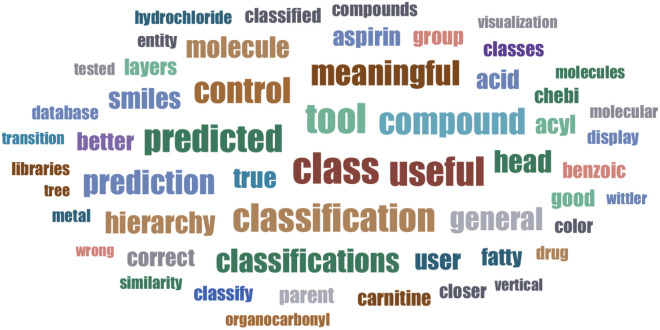
Word cloud of participant responses to survey questions.

A total of 12 participants engaged in the survey, testing a diverse range of SMILES inputs and offering useful suggestions on both the user interface and tool functionality (Fig. 7, Word cloud).

Out of these, 9 participants (75%) rated the classifications as either correct or partially correct as well as appropriate for the provided input molecules. In contrast, one solitary user (8%) noted inaccuracy, while two participants (17%) refrained from specifying accuracy level in their responses. These were instances of reported missing or inaccurately assigned classifications:

● *“Yes, they are true but do not describe the most important point. This is a transition metal complex! Hence, transition metal should come as classification as well.”*

● *“Yes, but only one classification was made and many others are equally true, for example**d**-galactose, cerebroside, β-galactosylceramide.”*

● *“Four answer provided. Three are correct. One is not the organocarbonyl was not correct. I suspect it called the amide a as an organocarbonyl.”*

Considering the correct classifications, 7 participants (58%) acknowledged the classification's chemical significance, either fully or to a partial extent. Conversely, 2 users (17%) expressed no utility in the classification, while 3 users (25%) did not further elaborate their response. However, a recurring theme was that the classifications were reported as being overly general, highlighting the need for a finer level of granularity:

● *“Yes they are meaningful if someone want to look at the general classes but if users want to go into a more detail level of granularity, especially for natural product classes, then ClassyFire may be a better option.”*

● *“I see no reason why the one classification that was made is more meaningful than the other possible classifications.”*

● *“I tested several lipids and most of the time the class predicted was a close parent. However, there were cases in which the class predicted by the tool was true but too general […].”*

Regarding the visualisation of a subset of the ChEBI hierarchy, only 2 users (17%) reported it as being valuable. In contrast, 5 users (42%) abstained from answering this question, while the other 5 users (42%) found the visualization unsuitable for their purposes. Their feedback highlighted the superiority of other established software and suggested future improvements such as a vertical orientation display and the inclusion of labels to enhance the interface design:

● *“It is useful. I would recommend to turn the display of the hierarchy into a vertical tree marking the root class in another color such as red, and the molecule been classified in a color that the user can clearly see where is the instance/molecule located in the hierarchy. To read the labels of the two first parent layers would also increase clarity, so I would make them static and readable in the graph.”*

● *“Not really [useful] – none of the names can be seen without mousing over, so it cannot be scanned and taken in. I also prefer vertical orientation.*

● *“Only first nodes, after node organic molecular entity I think it is no longer useful. I guess it depends on what each user is looking for.”*

Moreover, for the attention clusters, 3 users (25%) detected disparities in attention allocation among different attention heads, with one conjecturing that this variance might stem from layer alterations:

● *“This seems to vary by altering layers, and HEAD assignments, so I'd suggest seeing the aspirin SMILES prediction by yourselves.”*

Conversely, the remaining 9 users (75%) either deemed the function unhelpful or refrained from assessing it due to a lack of expertise:

● *“I don't understand how to interpret this visualization. What is a head?”*

● *“I do not understand this well enough to answer.”*

Collectively, suggestions for enhancing the tool encompassed several areas, including the need for improved accuracy and improvements to the interface design:

● *“I think it would be easier if a user adds the SMILES and the tool automatically predicts the class instead of the user clicking on the save and predict class buttons first.”*

● *“Putting in a large group of structures and clustering them in meaningful ways would be useful for us.”*

● *“Thanks for working on this much needed tool. I would suggest to adjust it for closer parents, the higher levels can be drawn from the hierarchy.”*

As discussed in Section 2.1, as a direct response to the consistent user feedback that the classifications provided by Chebifier were often correct, but too general, we developed Chebifier 1.1, which – compared to version 1.0 – increases the coverage of ChEBI by 56% without significant changes in accuracy. Since the additional classes are all lower in the taxonomic hierarchy of ChEBI than previously covered classes, Chebifier 1.1 is able to provide more specific classifications than the earlier version and thus addresses a major concern raised by our users. We further illustrate the improvement in the specificity of the predictions in the next sub-section.

### 3.4 Evaluating the specificity of model predictions

**Fig. 8:**
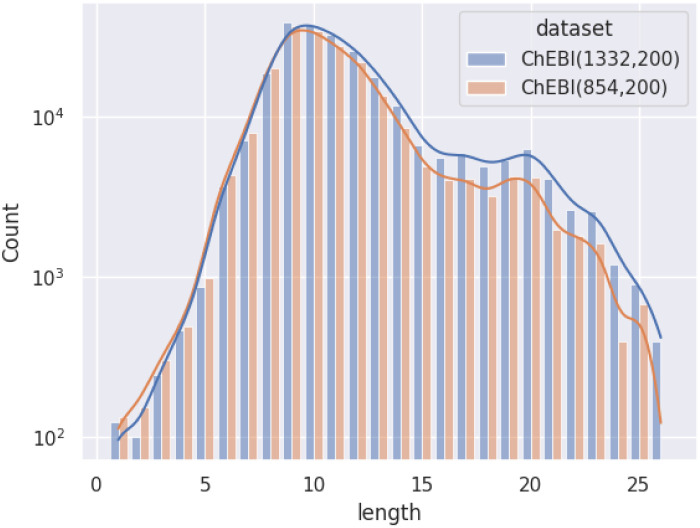
Distribution of path lengths (log scale) amongst correct non-redundant predictions on molecular entities in the test set as achieved by the weighted models on both datasets.

Our evaluation has shown that the increase in target classes does indeed lead to more predictions of classes further down in ChEBI's hierarchy. Fig. 8 shows the average path lengths of predicted classes to the *chemical entity* (CHEBI:24431).

**Fig. 9:**
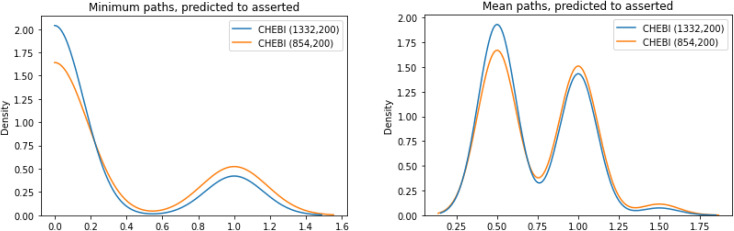
Minimum (left) and mean (right) paths lengths between the asserted parent for a SMILES and the predicted parent in the two datasets.

For each of the molecules in the test set of *ChEBI*^1332^_v200_ and *ChEBI*^854^_v200_, we evaluated the path lengths from the directly asserted parent in the ontology to the predicted parents in the calculated model predictions. The best case is that the distance is 0, *i.e.*, that the predicted parent class is the same as the asserted parent class. Fig. 9 shows the distribution of path lengths between classification parents and predicted parents in the two datasets.

To quantify the change of specificity between the different models, we also considered the subsumptions between predicted classes as shown in Table 3. For a given molecule and a correct prediction *C*_1_ by the *ChEBI*^1332^_v200_ model, all correct predictions of the *ChEBI*^854^_v200_ model for the same molecule are considered. If the *ChEBI*^854^_v200_ model predicts a subclass *C*_2_ of *C*_1_, then its prediction is considered more specific. If the *ChEBI*^854^_v200_ model predicts a superclass *C*_2_ of *C*_1_, then its prediction is considered less specific. In case both models agree on their prediction, their predictions are equally specific. In the last case, the *ChEBI*^854^_v200_ model makes no prediction that is in the same taxonomic branch as *C*_1_.§§More specifically, the *ChEBI*^854^_v200_ model does not correctly predict some class *C*_2_ such that *C*_2_ is comparable to *C*_1_ with respect to the partial order provided by the taxonomic structure of ChEBI. In this case, the predictions of the two models are considered as not comparable. As Table 3 shows, the predictions of the *ChEBI*^1332^_v200_ model are more specific in 21.69% and less specific in 8.43% of the cases compared to the *ChEBI*^854^_v200_ model. However, in most cases there is no difference between the models.

**Table 3:** Analysis of specificity between predictions made by the systems trained on *ChEBI*^1332^_v200_ and compared with *ChEBI*^854^_v200_

	More specific	Less specific	Equally specific	Not comparable
N	5021	1951	14 448	1724
%	(21.69%)	(8.43%)	(62.43%)	(7.45%)

The three different approaches to evaluate specificity lead to a consistent result: in comparison to its predecessor Chebifier 1.1 provides more specific predictions. However, its increase of coverage of ChEBI by 56% does not translate into an equally large increase in the specificity of its predictions. We will discuss the reasons in Section 4.2.

## 4 Discussion

Semantic resources such as ontologies are key for assigning meaning and interpretation to data, which in turn drives discovery research. Yet the maintenance of these knowledge resources is a challenging task that requires significant contributions from domain experts, making it slow and labour intensive. The pace of scientific discovery, however, is continuously accelerating, driven in part by the growing integration of artificial intelligence in all research areas.^22^ To support the evolution of ontologies at sufficient pace and scale requires semi-automated tools and approaches that are faithful to the existing domain knowledge and ways of thinking about scientific content, while also having the potential to learn directly from data. Our approach offers a strategy to navigate this tension in a sustainable ‘AI in the loop’ fashion, with the potential to be kept up to date automatically and significantly accelerate the maintenance of ontology-based knowledge resources in chemistry to keep up with the pace of discovery.

### 4.1 Related work

Our work builds on prior work we have conducted to lay the foundations of our approach.^10,12,14^ In these prior works, we established that Transformer architectures offered the best performance for the multi-class chemical ontology prediction problem, however, we were only able to predict a rather limited set of 500 ChEBI classes. The current work extends that prior work to a much larger set of ChEBI classes and introduces a novel weighting scheme to improve classification specificity as well as a graphical user interface through which users can interact with the model. Furthermore, we tested the user interface in a user study.

We consider the approach we present in this paper in the context of other approaches that aim to extend knowledge resources in chemistry automatically. There are a range of different strategies to include expert knowledge into a data-driven system to support ontology development. One example of a tool that is able to automatically classify chemical structures into chemical classes is ClassyFire.^23^ ClassyFire consists of a large set of structure-based patterns (captured in SMARTS, an extension of SMILES for pattern representation^24^) that allow for a rule-based classification of chemicals. ClassyFire^23^ is the state of the art tool for structure-based chemical ontology classification. Given the volume of user submissions to the database, the development process of ChEBI in many cases already uses ClassyFire to generate a first proposal for a classification of new chemical structures, particularly when a large set of structures is requested in a ‘bulk submission’. However, ClassyFire does not use the ChEBI ontology directly, but rather offers its own chemical classification ChemOnt, which is a purely structure-based chemical taxonomy consisting of 4825 classes. In its underlying ontology, ClassyFire's conceptualisation of the domain of chemistry differs from that of ChEBI, and thus, the mapping from ClassyFire's representation to ChEBI's results in a loss in precision due to differing approaches for treating conjugate bases and acids. As a result it is not straightforward to directly compare our approach to ClassyFire's. In our previous research, we compared an earlier version of our approach to ClassyFire's predictions and found that our strategy was on average able to make more precise predictions in ChEBI.^12^

Our approach is functionally similar to ClassyFire in the sense that it enables the automatic classification of chemicals based on their chemical structure (as represented in SMILES). However, there are major differences. First, Chebifier predicts ChEBI classes directly, there is no other taxonomy involved, thus no alignment step. Second, ClassyFire classifies chemicals with the help of roughly 9000 rules, which are manually maintained by its developers and form a fixed part of its implementation as a software library. In contrast, Chebifier uses an artificial neural network, which is trained based on ChEBI data.

Furthermore, rule-based approaches such as ClassyFire are tailored to a specific use case and ontology. An extension of the scope of such a tool requires additional investment of time and effort for manually adding new patterns and rules, which is a modelling process that is not dissimilar to ontology development. Other approaches therefore aim to circumvent this manual process. However, the specific domain knowledge is still required and must therefore be obtained from other sources.

Many approaches to (semi-)automatic ontology extension process large corpora of relevant domain literature as the source of the required domain knowledge, to extract important entities. A survey^25^ of existing ontology learning mechanisms shows that a majority of them follows a common pattern. The first step is the extraction of entities that are relevant for the target domain, often based on statistical methods such as term frequencies^26,27^ or clustering methods. In a next step, relations between these extracted entities are extracted using techniques such as association rule learning^28,29^ and inductive logic programming.^30,31^ These literature-driven approaches however suffer from one common drawback: they assume that the literature reflects a general consensus amongst experts on the conceptualisation of their domain. Such a consensus however often does not exist, and building a consensus is often what makes the ontology development time-consuming in the first place.^13^ A system that is supposed to successfully extend an ontology is therefore required to first understand the underlying design principles that an ontology is based on. One way to achieve this is to use the ontology itself as training data for a model. Such ontology-based training has been shown to be useful in chemistry based ontology extension^10,14^ and builds the foundation for the presented Chebifier tool. It was also shown that this kind of training provides domain-relevant knowledge beyond the scope of the ontology.^32^ A similar approach based on large language models has been proposed to classify entities within the DOLCE upper-level ontology,^33,34^ and a graph-based representation learning strategy has been used to suggest classifications in the SNOMED clinical vocabulary.^35^

A benefit of our approach over and above enabling chemicals to be classified into an ontology, is that it encodes the knowledge from the ontology into a computable format that is then able to be applied through transfer learning to improve other classification tasks. For example, we have shown that ontology pre-training improves performance of a prediction model in predicting chemical toxicity, even though the pre-training did not include any biological information directly relevant to the toxicity prediction task.^32^ This illustrates the broad-ranging importance of incorporating domain knowledge into data-driven discovery methods. A similar approach in the domain of proteins is OntoProtein,^36^ which uses the Gene Ontology to augment the information available for a protein language model.

### 4.2 Limitations

While Chebifier has delivered promising results, the approach nevertheless still has some limitations which we note here.

One limitation is that the ChEBI axioms are not taken into account. The simplest axioms are subclass and disjointness axioms, more complex (and more rare) axioms concern parthood relations and functional parents. As described in the next subsection, in future work we plan to adopt a neuro-symbolic approach to take these axioms into account in the classification process. This can be done by computing a degree of axiom violation and feeding this as a semantic component into the loss function.

Another limitation of the tool relates to the specificity of the predictions. In our user study, users reported that they often found the classifications provided by Chebifier to be too general. As a result, we developed Chebifier 1.1, which – compared to version 1.0 – increases the coverage of ChEBI classes by 56%. As we discussed above, this increase was achieved without significant changes in accuracy. The increase in coverage can already be expected to improve the utility of the tool for a range of use cases. However, it would be additionally beneficial if it were to have dramatically increased the specificity of the model predictions. Unfortunately, although we did observe an increase in the specificity of model predictions across a range of metrics, the observed increase of prediction specificity was only moderate. This result can largely be attributed to two main causes.

First, *ChEBI*^1332^_v200_ extends *ChEBI*^854^_v200_ with classes that have between 50 and 99 examples (*i.e.*, classes that have between 50 and 99 members, that is subclasses which are annotated with SMILES in ChEBI). *ChEBI*^1332^_v200_ is trained on 129 187 molecules. As a result, only between 0.039% and 0.077% of molecules in the dataset are members of any given class amongst those that are in *ChEBI*^1332^_v200_ but not in *ChEBI*^854^_v200_. Hence, any one of these additional classes is irrelevant for the majority of classification tasks. In fact, only 18.79% of all molecules in the dataset are a member of any of the 478 additional classes. Thus, extending *ChEBI*^854^_v200_ by 478 of these relatively rare classes can only be expected to have a limited impact on the overall performance. Second, since Chebifier is trained based on datasets generated from ChEBI's taxonomy, it learns to imitate ChEBI. And because ChEBI contains a lot of examples of chemical entities that are classified on a quite high level, Chebifier imitates that lack of specificity. For example, *lactam* (CHEBI:24995) has 7328 direct subclasses. Thus, while ChEBI provides a taxonomy that enables classification of different types of lactam, given the vast number of molecules that are directly classified as lactam by ChEBI, it is difficult for a machine learning model to learn the subclasses of lactam. Lactam is not unique, *e.g.*, azamacrocycle (CHEBI:52898, 7246 direct subclasses) and peptide (CHEBI:16670, 6199 direct subclasses) are two other examples for classes that are used to classify molecules on a high level of generality. Note that these general classifications are usually not the result of ChEBI's manual curation process, but the result of semi-automated processes that are already adopted in the development process, largely using the ClassyFire tool. A specific mitigation strategy for this problem in the future will be to exclude these overly general classes from the training data and subsequently re-classify all the children of the classes in a targeted ontology specificity improvement step. Alternatively, it may be possible to exclude automatically created content from ChEBI to create a smaller but more accurate training dataset. However, while it is not optimal to train our model using content that was automatically added to ChEBI rather than only the 3-star fragment of manually assembled content, it is potentially somewhat challenging to extract only the 3-star fragment of ChEBI as an ontology, as the star rating is published for classes but not for relationships between classes, and it is necessary to extract a fully connected ontology which will preclude bypassing, for example, mid-level classes that are not 3-star. Due to the associated complexity, we have used the full version of ChEBI for the current work but in future work we do plan to develop an algorithm to filter out as much of the automated part of ChEBI as possible while preserving the connected ontology hierarchy.

An alternative strategy in the future to further increase the specificity of predictions would be to include classes with even fewer examples (*e.g.*, classes with at least 25 subclasses that are annotated with a SMILES), although there are additional challenges to training models to learn from such a small number of examples. In general, as the development of ChEBI progresses and ChEBI is improved by providing more examples of more specific classifications, Chebifier's ability to provide more specific classifications will also in turn improve.

A further limitation relates to the use of a sequence-based encoding for the input chemical structures. We have observed that while the average performance of the approach is good, the model is weak in predicting classes that are defined based on complex ring structures, in particular aromatic structures. Future work will evaluate alternative representation strategies for ring structures and consider building ensembles of different approaches to balance the strengths and weaknesses of different encoding strategies.

While neural networks are black boxes in general, we have offered a rudimentary form of interpretability by visualising the activations of the transformers' attention heads. However, our user study revealed that the majority of users did not find this visualisation useful in its current form. Thus, to address this limitation, in future work, we plan to use other explanation mechanisms that are closer to the intuitive human understanding. One option is to visualise aggregated attention directly on the atoms of the molecule, a strategy that we illustrated previously.^10^ However, the best way to aggregate attention to provide the maximally meaningful visualisation has not yet been determined. We plan to explore automatically selecting attention sub-components with maximal semantic relevance for aggregated display. Moreover, we plan to train separate (possibly simplified) networks for each class in order to be able to focus on attentions that are relevant for the selected class. Finally, as a different interpretability strategy altogether we plan to explore traditional mechanisms such as decision trees.

### 4.3 Future work and use cases

The main use case of Chebifier is to enable the automatic classification of chemicals within ChEBI ontology classes. For this purpose users may use our online server or download the Chebifier software library and run it as part of their own infrastructure. Thus, the use of Chebifier does not necessarily require public submission of data to an online server, which for some users (*e.g.* pharmaceutical companies) may be impossible.

Another use case of Chebifier is as an auxiliary tool for ontology developers. ClassyFire is already used as part of ChEBI's ontology development process, and we hope that in the future Chebifier will be equally useful to support ChEBI developers in their important work.

A more advanced use case in the context of data-driven discovery is that the Chebifier model can be further fine-tuned to enable the domain knowledge which the model has learned from the ontology to be harnessed to improve performance for other, more specific prediction and discovery tasks. We have previously shown that this approach has value for example in toxicity prediction,^32^ and a similar approach can also be used to extend the Chebifier model for better performance in a sub-domain of interest. For example, if a task requires an improved and more fine-grained classification of lipids than Chebifier can provide out of the box, then it is possible to further fine-tune Chebifier's model by providing a set of examples for the kinds of lipids that are relevant for the task. The resulting model will be able to automatically classify lipids more reliably and more precisely.

In the future, we aim to improve the performance of Chebifier by employing neuro-symbolic architectures (*e.g.* using logical neural networks^37^) in order to harness the additional information contained in ontology axioms and use that additional information (*via* a semantic loss function) for improvement of the classification. Furthermore, we plan to evaluate whether other representations of molecular structures than SMILES, *e.g.*, graph neural networks, may improve prediction performance. We are also aiming to extend the prediction model with an ensemble of heterogenous approaches. One of the main limitations of our approach is that it requires a minimal number of individuals in order to predict a class with sufficient reliability. The leviate this, we aim to use more symbolic approaches to predict classes that are lower in ChEBI's hierarchy. SMARTS is a more expressive extension of SMILES that allows the description of complex patterns. While there are no sufficient datasets, yet, to train our system as-is, we plan to generate formal descriptions (*e.g.* using SMARTS or even first-order^38^ or higher-order^39^ logic) of ChEBI classes either manually or semi-automatically.

Finally, we plan to extend the online web tool to address the remaining requests from the user study including enhanced visualisations. At the same time, we plan to add a facility to collect expert feedback on the provided Chebifier classifications. This data can then also be used to improve the model itself (a ‘human in the loop’ strategy) and in the long run, enable Chebifier-generated classifications to be tuned to be better suited for direct inclusion into ChEBI, further accelerating the continuous learning and discovery cycle.

## 5 Conclusions

We offer a tool and API for the semi-automation of chemical ontology development and the automated application of the ontology to annotate novel chemical structures. The tool and API are freely available and licensed permissively. We evaluated the approach with several strategies and tested the usability and acceptability in a user study.

Finding the right approach to harness existing knowledge and build data-driven models that in turn are able to improve knowledge resources is a key ingredient to accelerate the positive feedback loops of data-driven discovery and reduce waste in research. We believe that our approach has broad relevance to accelerate a number of different downstream tasks.

## Data availability

The project is developed as open source which is available on GitHub at https://github.com/ChEB-AI/Chebifier for the web tool and https://github.com/ChEBAI/python-chebai for the API, with a permissive license for re-use. The web tool is openly accessible online at https://chebifier.hastingslab.org/. Source code, input data and model files are also available as manuscript ESI† in a citeable archive on Zenodo. See DOI: https://doi.org/10.5281/zenodo.10247092.

## Author contributions

Martin Glauer: data curation (lead); formal analysis (equal); investigation (equal); methodology (equal); software (lead); validation (equal); visualisation (equal); writing – original draft (equal); writing – review and editing (equal). Fabian Neuhaus: conceptualization (equal); supervision (equal); writing – original draft (equal); writing – review and editing (equal). Simon Flügel: data curation (supporting); formal analysis (supporting); investigation (equal); software (supporting); validation (equal); writing – original draft (equal); writing – review and editing (equal). Marie Wosny: investigation (equal); writing – original draft (equal); writing – review and editing (equal). Till Mossakowski: funding acquisition (equal); supervision (equal); project administration (equal); writing – original draft (equal); writing – review and editing (equal). Adel Memariani: software (supporting); writing – original draft (supporting). Johannes Schwerdt: software (supporting); writing – original draft (supporting). Janna Hastings: conceptualization (equal); formal analysis (equal); funding acquisition (equal); investigation (equal); methodology (equal); project administration (equal); supervision (equal); visualisation (equal); writing – original draft (equal); writing – review and editing (equal).

## Conflicts of interest

There are no conflicts to declare.

## Supplementary Material
